# The role of psychosocial working conditions on burnout and its core component emotional exhaustion – a systematic review

**DOI:** 10.1186/1745-6673-9-10

**Published:** 2014-03-14

**Authors:** Andreas Seidler, Marleen Thinschmidt, Stefanie Deckert, Francisca Then, Janice Hegewald, Karen Nieuwenhuijsen, Steffi G Riedel-Heller

**Affiliations:** 1 Institute and Policlinic of Occupational and Social Medicine (IPAS), Technische Universität Dresden, Dresden, Germany; 2 Institute of Social Medicine, Occupational Medicine and Public Health (ISAP), Leipzig, Germany; 3 Coronel Institute of Occupational Health, Academic Medical Centre, Amsterdam, Netherlands

**Keywords:** Psychosocial, Working conditions, Emotional exhaustion, Burnout, Systematic review

## Abstract

**Aims:**

To analyze the association between psychosocial working conditions and burnout and its core component emotional exhaustion, a systematic literature review was undertaken including cohort studies, case–control studies, and randomized controlled trials.

**Methods:**

The literature search in Medline and PsycInfo was based on a defined search string and strict exclusion and inclusion criteria. Evaluation of the 5,599 initially identified search hits by two independent reviewers and a detailed quality assessment resulted in six methodologically adequate cohort studies considering the relationship between psychosocial working conditions and burnout (one study) as well as the burnout core component emotional exhaustion (five studies).

**Results:**

The results of our systematic review point to a relationship between psychosocial working conditions and the development of emotional exhaustion/burnout. Particularly high job demands seem to play a role in the development of emotional exhaustion. However, strong intercorrelations between workplace factors, as a matter of principle, make the identification of a single psychosocial workplace factor (being associated with an especially high or low risk of burnout) difficult.

**Conclusions:**

Multidimensional approaches including reduction of work demands, enhancement of decision latitude and improving the social climate might be promising for preventing burnout and emotional exhaustion. However, methodologically adequate intervention studies are urgently needed to prove the effectiveness of workplace interventions.

## Introduction

Contemporary workplaces are characterized by increasing work pace, increasing expectations for self-actualization, increasing reliance on interpersonal coordination in the execution of work tasks, increasing pace of change, and increasing job insecurity. Against this background, the influence of psychosocial working conditions on mental health, but also on cardiovascular and musculoskeletal disorders is under discussion [1-5].

On a general level, mental disorders are characterized by multifactorial genesis in which genetic as well as environmental factors play an etiologic role [6]. In this context, it is increasingly recognized that working conditions are also important determinants of mental health as it can have both beneficial and harmful influences on the individual worker’s health status [7,8].

In the mid-1970s, the investigation of the so-called burnout phenomenon was established at first with respect to service professionals [9]. Since then the burnout syndrome, particularly its relationship to prolonged interpersonal work-related stress has been controversially discussed from various perspectives (e.g. in the fields of psychiatry, psychosomatics, occupational medicine and health psychology) [10-12]. Maslach et al. divided the burnout syndrome into three dimensions: *emotional exhaustion* (feelings of being “empty”) as the core component, *depersonalization* (negative, cynical attitude toward the work or the recipients) and *reduced personal accomplishment* / *professional efficacy* (negative evaluation of the achievements at work) as additional components [10,13-16]. Pines et al. [17] defined burnout not only as a state of emotional, but also physical and mental exhaustion in long-lasting emotionally demanding situations. It is becoming apparent that especially emotional exhaustion can be understood as the core component of burnout. In understanding burnout as a stress-related adaptive process, Nil et al. [11] described the development of burnout “… as going through several phases, from increased working efforts to cope with external demands, which can lead to mental and physical exhaustion and demotivational affective states, and on to psychosomatic complaints and finally depressive state” (p. 72). Burnout is not regarded as a medical or psychiatric diagnosis as defined by the ICD-10 or DSM-IV manuals. In the ICD-10 burnout has the status of a residual category [18]: “Z73.0: problems associated with difficulties in coping with life, that affects the health and leads to a demand of health services”. The differential diagnosis of burnout to clinical diseases is maldefined and includes ruling out other diseases with similar clinical components, e.g. depression (ICD-10 diagnosis code F32.), neurasthenia (F48.0, also fatigue syndrome), chronic fatigue syndrome / benign myalgic encephalomyelitis (G93.3), insomnia (F51.0) or posttraumatic stress disorder (F43.1) [19].

Although there are currently no consistent standardized diagnostic criteria for burnout, estimates on the prevalence of burnout were made in some studies, specifically related to specific occupational groups. Research findings about the prevalence of burnout exist primarily for service-related professions (e.g. teachers, nurses or physicians). Depending on the professional group, the prevalence estimates differ significantly from each other. Nil et al. [11] investigated the status quo of burnout prevalence rates, estimating that between 3.5% and 12% of Swiss primary care doctors [20], and up to 50% of emergency room workers were affected [21,22]. Arora et al. [23] reported a burnout prevalence among orthopaedic surgeons between 50-60% and among surgeons in general between 30-40%. Of course the determinability of the prevalence for a phenomenon that is not consistently defined might be questioned. However, burnout affected persons clearly suffer from the symptoms of this syndrome [19]. Moreover, burnout potentially constitutes a developmental state of a depressive disorder [19]. Therefore, the high – although inconsistent – estimations on the prevalence of burnout point to a high public health impact.

In general the burnout “diagnosis” is based on self-reported screening instruments. The Maslach Burnout Inventory (MBI) including its specific versions and subscales (e.g. MBI-General Survey - GS) is the most commonly used burnout measure, which is applied in most of all studies concerning burnout [24,25]. Other established burnout questionnaires are the Copenhagen Burnout Inventory (CBI [26]), the Oldenburg Burnout Inventory (OLBI [27]), the Shirom Melamed Burnout Measure (SMBM [28]), Gillespie-Numerof Burnout Inventory (GNBI [29]) or the Tedium Measure (TM [17,30]). Given the different definitions of burnout in these listed questionnaires, the comparability of the results is somewhat limited.

Existing systematic reviews on the psychologic effects of psychosocial working conditions mainly focused on depression [31,32], mental disorders in all [33], and stress-related disorders [34]. There are also some systematic reviews that focus on the relationship between (inter alia) work-related factors and burnout [35-41]. However, the mentioned systematic reviews on burnout address very specific occupational groups: medical residents [36,37], pediatric oncology staff [38], correctional officers [35,40], elderly care staff [39], or nurses [41]. Thus, the generalizability of the main results to other occupational groups might be restricted. In addition, a quality assessment of included studies, which is an essential work step preparing a systematic review, was unfortunately conducted in only three of the fore cited previous systematic reviews [37,38,40]. In contrast to the topic of psychosocial working conditions and cardiovascular diseases – where several longitudinal studies can be found – most studies on burnout/emotional exhaustion apply a cross-sectional approach. All mentioned reviews therefore included cross-sectional studies which might have introduced substantial cause-and-effect bias. The few reviews that also included some longitudinal studies found limited or unclear evidence for the relationship between psychosocial working conditions and burnout: In the review published by Thomas [36], only one of 12 included observational studies on the association between psychosocial working conditions and burnout applied a longitudinal design; Prins et al. [37] identified three longitudinal studies with inconsistent findings concerning the impact of long working hours.

The aim of this systematic review is to provide a comprehensive overview about the effects of psychosocial working conditions on the development of burnout and its core symptom, emotional exhaustion. In contrast to the mentioned previous reviews the provided systematic review on psychosocial working conditions and burnout excludes cross-sectional designs; moreover, this systematic review allies a comprehensive quality assessment with standardized checklists.

## Material and methods

### Study design

This systematic review is part of a comprehensive review on psychosocial working conditions and mental health, including emotional exhaustion, burnout, depression, anxiety disorder, and somatoform disorder according to ICD-10, DSM-VI. As we conducted a combined search for all of the outcomes mentioned above, the paragraphs Eligibility criteria and Data abstraction are related to all of the outcomes, not to emotional exhaustion and burnout alone. The presented systematic review was performed in accordance with the PRISMA statement [42].

### Eligibility criteria and identification of articles

A systematic electronic literature search was done in (1) Medline via the PubMed interface and (2) PsycInfo via EBSCO host interface from inception (Medline 1948, PsycInfo 1966) until November 30, 2013. The search strings which included the search strategy published by Mattioli et al. [43] are given in Additional files 1 and 2. We limited the systematic electronic search to English and German articles with abstracts. We included all randomized controlled trials (RCT), cohort studies or case–control studies.

Initially, titles and abstracts of the studies were screened by two independent scientists (SD, FT) to eliminate studies that were clearly unrelated to the a priori defined research questions. The included papers were assigned to the specific research questions. There was a rather good inter-rater reliability between the two reviewers (agreement = 92%, Cohen’s kappa = 0.50). The reference lists of relevant key articles and narrative as well as systematic reviews were screened to identify further relevant studies.

Subsequently, the full texts of the remaining studies were thoroughly and independently examined by two reviewers (SD, FT) to determine if the inclusion criteria for this specific review were met. The eligibility criteria were specified as follows (see Table 1):

– Population: working population, age: 17 + .

– Exposure: psychosocial working conditions.

– Outcome: burnout and emotional exhaustion.

**Table 1:** Eligibility criteria

	**Inclusion criteria**	**Exclusion criteria**
**P**opulation	working population, age: 17+ (no age limit because of manifestation of effect in old age)	age <17 years, unemployed subjects
**E**xposures	*Psychosocial work stressors*:	chemical (e.g. solvents, lead, manganese) or physical factors (e.g. noise, electromagnetic fields), physical requirements of the job, not-work-environment-related stressors (e.g. family caregiving), vocational training or study
	stress, mental load, work load, effort, reward, job strain, job demand, job control, shift work, time pressure, job insecurity, institutional changes like down-sizing or merger, social support/ mobbing, bullying, leadership style, climate, work-related justice	
**O**utcome	burnout and emotional exhaustion	physical disorders/ impairments, chronic fatigue syndrome, post-traumatic stress disorder, psychological distress, psychiatric distress, psycho-social well-being, mental health or psychiatric morbidity in general
**O**utcome **M**easure	valid self-rating scales, clinical diagnosis with/ without structured interview, secondary data	unvalidated instruments (e.g. single items)
**D**esign	cohort, case–control, RCT	all others
**P**ublication **T**ype	articles in journals and with available abstract	books, book chapters, book reviews, comments, corrections, editorials, introductions, forewords, letters, replies, dissertations

According to Semmer and Mohr [44] the exposure was classified into three categories: work organization, work task and social condition. As this systematic review strictly focuses on psychosocial working conditions, we excluded work-related conditions that are associated with private or family life, e.g. work-privacy conflict or work-family conflicts. We nevertheless would like to underline that work-privacy conflicts cannot be regarded as exclusively privacy-related factors: work-privacy conflicts are related to work in the sense that unfavorable working conditions lead to conflicts in the private or family life. Emotional exhaustion could be measured by burnout-subscales (e.g., EE scale of the Maslach Burnout Inventory - MBI) or by multi-item measures; single-item outcome measures were excluded from our systematic review. All established measurement instruments (e.g. MBI, MBI-GS, OLBI) are self-administered questionnaires.

The exclusion of studies at this stage was discussed in consensus teleconferences; the reasons for exclusions (e.g., improper study designs – i.e. narrative review or cross-sectional study, duplicate publications, irrelevant populations, poorly defined outcomes) were documented for each paper.

### Data abstraction

Data abstraction from the included articles and study quality assessment were done independently by two reviewers (MT, FT) and discussed in consensus teleconferences. The data abstraction form included information on relevant study characteristics such as study design, study region, source population, number of participants, participant characteristics (age, sex), exposure or intervention, potential misclassification of exposure, duration of follow-up, outcomes, potential misclassification of outcomes, and funding. The mentioned study characteristics of the relevant studies were entered into evidence tables (Table 2).

**Table 2:** **Methodologically adequate studies on the association between psychosocial workload and emotional exhaustion**/**burnout**

**First author, publication year**	**Study region**	**Study design**	**Population**				**Exposure**	**Outcome**	**Results**
			**Branch/Occupation; no. of companies**	**No. of subjects *(response rate, mean age)***	**Time of baseline examination**	**Follow up *(mean, range)***			
Ahola & Hakanen 2007 [45]	Finland	Follow-up study	Finnish dentists (members of Finnish Dental Association)	2,555 Finnish dentists (71% response of all Finnish dentists)	2003	2006 (3 years), loss to follow-up 22%	Job strain (Job Content Questionnaire - JCQ)	Burnout (Maslach Burnout Inventory - MBI)	Subjects free of burnout at baseline, adjusted for depression at baseline:
									**Men**
									job strain: OR=22.3 (95% CI 5.1-98.1)
									**Women**
									Job strain: OR=4.0 (95% CI 2.0-8.0)
									Subjects free of burnout but with depressive symptoms at baseline:
									Job strain: OR=2.2 (95% CI: 1.4-3.4)
									Subjects free of depression (measured by Beck Depression Inventory - BDI) at baseline, adjusted for baseline burnout:
									Job strain: OR=1.8 (95% CI: 1.04-3.1)
Janssen and Nijhuis 2004 [46]	Netherlands	Follow-up study	45 companies	5,256 employees (response rate 45%, mean age 42.3±8.5 years)	1998	1 year, loss to follow-up 20%	(1) Psychological demands (JCQ, Dutch version)	Burnout-subscale emotional exhaustion of MBI-GS (General Survey)	Reduced emotional exhaustion:
									(1) Decreased job demands: β=-0.16, p<0.001
							(2) Decision latitude (JCQ, Dutch version)		
									(2) Increased decision latitude: β=0.07, p<0.001
							(3) Social support (JCQ, Dutch version)		
									(3) Increased social support: β=-0.07, p<0.001
Langballe et al. 2011 [47]	Norway	Follow-up study	physicians	n=291 female physicians (response rate 74%, mean age 41.8±9.9 years), n=232 male physicians (response rate 64%, mean age 48.1±10.9 years)	2005	2 years, loss to follow-up 21% (women) and 26% (men)	(1) Perceived workload (3-item scale, based on the Job Stress Questionnaire - JSQ)	Burnout-subscale exhaustion of Oldenburg Burnout Inventory (OLBI, Norwegian version)	**Women**
									(1) High workload (follow up): β=0.17, p<0.01
							(2) autonomy (4-item scale, based on the Job Stress Questionnaire - JSQ)		(2) High autonomy (follow up): β=0.07, n. s.
							(3) no. of hours worked per week		(3) Working hours (baseline): β=0.01, n. s.
									**Men**
									(1) High workload (follow up): β=0.31, p<0.01
									(2) High autonomy (follow up): β=0.22, p<0.001
									(3) Working hours (baseline): β=0.03, n. s.
	
Lorente Prieto et al. 2008 [48]	Spain	Follow-up study	23 secondary schools	N=274 teachers (response rate 81%, mean age 40±7.0 years, 43% men)	n.r.	8 months, loss to follow-up 43%	(1) quantitative overload (3-item scale, instrument n. r.)	burnout-subscale emotional exhaustion of MBI-GS	(1) Quantitative overload (baseline): β=0.12, p<0.05
							(2) mental demands (6-item scale, instrument n. r.)		Results for exposure no. (2)-(7) n. r., n. s.
							(3) emotional demands (7-item scale, instrument n. r.)		
									Women feel more exhaustion (β=0.11, p<0.05) at follow-up than men
							(4) role ambiguity (6-item scale, instrument n. r.)		
							(5) role conflict (8-item scale, instrument n.r.)		
							(6) autonomy (5-item scale, instrument n. r.)		
							(7) social climate (3-item climate scale of the FOCUS questionnaire [49]		
Taris et al. 2010 [50]	Nether-lands	Follow-up study	Dutch police officers	828 police officers, mean age 42.1±7.8 years, 85% men, response rate for baseline examination 53%	n.r.	1 year, loss to follow-up 57%	(1) job demands (4-items of the JCQ)	burnout-subscale emotional exhaustion of MBI-GS (Dutch version)	(1) High demands: β=0.08, p<0.01
									(2) Job control: n. s.
							(2) job control (9-item scale: 1 item from JCQ, 3 items from Dutch Stress Questionnaire, 5 items from the NOVA-WEBA questionnaire [51]		

Van Vegchel et al. 2004 [52]	Sweden	Follow-up study	Human services	2,255 human service employees, mean age 47.0±6.5 years, 41% men, response rate 76%	1997	1 year, loss to follow-up 29%	(1) Quantitative demands (4-item scale based on the JCQ)	Burnout-subscale emotional exhaustion of MBI (Swedish version)	Model 1:
									(1) High quantitative demands: β=0.12, p<0.01
							(2) Emotional demands (8-item scale)		(3) Low job control: β=-0.10, p<0.05
									(4) Low social support: β=-0.07, p<0.05
							(3) Job control (8-item scale, instrument n. r.)		
									Model 2:
							(4) Social support (7-item scale, instrument n. r.)		(2) High emotional demands: β=0.09, p<0.001
									(3) Low job control: β=-0.10, p<0.05
									(4) Low social support: β=-0.08, p<0.05

*Abbreviations*: *n. s.* not significant, *n. r.* not reported, *JCQ* Job Content Questionnaire; *MBI* Maslach Burnout Inventory, *MBI*-*GS* Maslach Burnout Inventory – General Survey.

### Critical appraisal of the literature (scoring/discussion of possible bias)

The studies were examined according to a combination of the criteria described by SIGN (Scottish Intercollegiate Guidelines Network [53] and CASP (Critical Appraisal Skills Programme of the British National Health Service Appraisal Tools [54]). The applied checklists are available online. Two reviewers (MT, FT) independently and systematically assessed the studies on a three-level scale (++, +, -) considering the internal/external validity and documented their results on an appraisal form. The study evaluation criteria are defined according to SIGN [53] as follows:

++ All or most of the criteria have been fulfilled. Where they have not been fulfilled the conclusions of the study or review are thought very unlikely to alter.

+ Some of the criteria have been fulfilled. Those criteria that have not been fulfilled or not adequately described are thought unlikely to alter the conclusions.

- Few or no criteria fulfilled. The conclusions of the study are thought likely or very likely to alter.

According to the SIGN criteria, studies were classified as of low methodological quality (-) when methodological weaknesses were believed to considerably influence the core results. The results of the critical appraisal were compared and discussed in teleconferences (MT, FT, AS), leading in all cases of diverging opinions to a consensus.

Depending on the homogeneity of the included studies, the results should as possible be pooled within a meta-analysis. To allow for a meta-analysis, we a priori determined that the considered study populations, definition and measurement of exposures and outcomes, study designs as well as the statistical procedures should be comparable. Otherwise, depending on the number of studies, subgroup analysis should be considered. To provide an informative basis, at least three studies should be available for one subgroup analysis.

## Results

**Figure 1:**
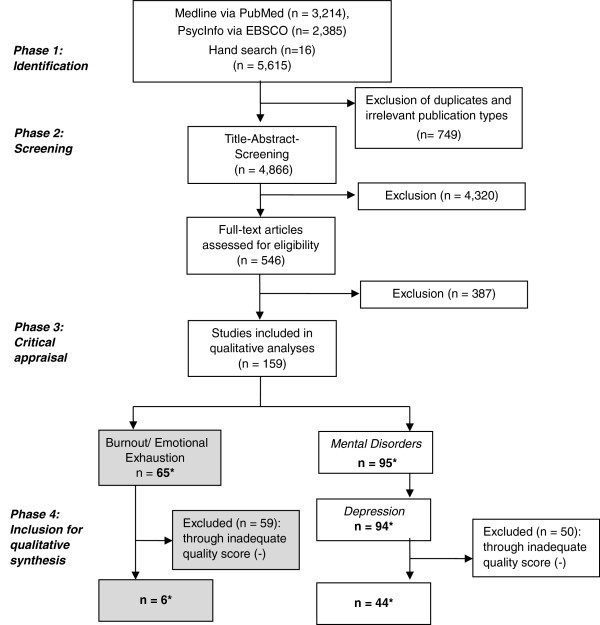
**Flow chart of study selection procedure for systematic review of burnout and mental disorders and psychosocial working conditions (according to the PRISMA statement**, **Moher et al. **[42]). *Note: One study reported about both burnout/emotional exhaustion and depression (duplication), the subject of this review is marked grey.

Of 5,599 initially identified search hits and 16 articles found by hand search, 749 publications were excluded by publication type (comments, chapters in books, dissertations) or as duplicates at this stage. The titles and abstracts of 4,866 citations were screened by two independent reviewers, and 4,320 articles were excluded after review. Of the resulting 546 articles, 387 were excluded after full-text review because they did not meet the eligibility criteria, so that a total of 159 remained (Figure 1), 65 for burnout / emotional exhaustion and 95 for mental disorders (one study reported about both burnout / emotional exhaustion and depression). After quality assessment, a further 109 publications were discarded due to an inadequate quality score (-). The main reasons for an inadequate quality score were as follows:

– To avoid selection bias, the study base has to be clearly defined. Ad-hoc samples or the recruitment of health conscious subjects do considerably affect the external validity of the study. Furthermore, the studies should have a sufficiently high initial response (of about 50% or more). Moreover, to avoid substantial selection bias, the loss to follow-up should be low. Studies with a loss to follow-up of 50% or more are prone to substantial selection bias, so they were classified as methodologically inadequate, even if other sources of bias were lacking.

– To avoid reverse causation, the exposure had to be measured at baseline.

– The study population should not suffer from the outcome of interest at baseline to allow the distinction between before-and-after effects. At least baseline mental health had to be taken into account in the analyses (by adjustment).

– Confounding should be adequately considered. As a minimum, age and gender had to be taken into account (e.g. by adjustment or stratification).

Additional file 3 gives a list of the studies that were excluded based on inadequate quality assessment; the concrete reasons for inadequate quality scores are given by Thinschmidt et al. [55]. Of the remaining 50 studies, six methodologically adequate longitudinal studies dealt with emotional exhaustion or burnout and were therefore included in this systematic review. The characteristics of these six cohort studies are summarized in Table 2 and described narratively below. All of these six studies fulfilled some of the above mentioned quality criteria (quality score +), there were no studies that fulfilled all or most of the criteria (quality score ++).

The follow-up study of Ahola and Hakanen [45] recruited 3,255 of 4,588 dentists employed in clinical work in Finland. 2,555 subjects (74% women) participated at a three-year follow-up in 2006. The study is characterized by a high initial response and a relatively low loss to follow-up. Burnout was assessed with the Maslach Burnout Inventory (MBI). This study is the only included publication that did not consider the different burnout subscales separately, but considered burnout as total score. Furthermore, this publication examined the potentially mediational relationships between burnout and depression under the condition of job strain (measured by the Job Content Questionnaire, JCQ). Using logistic regression models (all models adjusted at least for sex, age, marital status, baseline burnout and depressive symptoms) job strain at baseline was associated with burnout at follow-up among those participants who were free of depressive symptoms at baseline (OR adjusted for – inter alia – baseline burnout = 1.8, 95% CI 1.0-3.1 for each one-point increase in job strain), and also for those with existing depressive symptoms at baseline (OR adjusted for baseline burnout = 2.2, 95% CI 1.4-3.4). Odds ratios were remarkably higher among those free of burnout at baseline and differed between men and women: job strain predicted burnout stronger in men (adj. OR for each one-point increase in job strain = 22.3, 95% CI 5.1-98.1, adjusted for baseline depression) than in women (adj. OR 4.0, 95% CI 2.0-8.0, adjusted for baseline depression).

Using longitudinal data from the Maastricht Cohort Study on “Fatigue at Work” (n = 5,256 male and female employees, numbers of females and males not given), Janssen and Nijhuis [46] analyzed the effects of positive changes in perceived work characteristics on fatigue, but also on emotional exhaustion over the period of one year (1998–1999) in a heterogeneous working population including all sectors. The initial response was low, but loss to follow-up was only moderate. As the selection of the final sample is clearly described, the study was classified as methodologically acceptable despite of the low initial response. The study examined the influence of a positive change in job demands, decision latitude, and social support (measured by the JCQ) on a reduction in emotional exhaustion (measured by the MBI-GS, subscale EE; one-year-span). Hierarchical regression models were performed. Emotional exhaustion decreased with decreasing job demands, increasing decision latitude and increasing social support compared with a situation without changing work characteristics. The authors did not conduct gender-specific analyses.

In their two-wave panel study Langballe et al. [47] observed a random sample of 291 female and 232 male Norwegian doctors (from each of 500 subjects) over a follow-up-period of two years (2003–2005). The authors examined the associations between work characteristics (workload, autonomy, measured by a three- or a four-item scale based on the Job Stress Questionnaire, JSQ) and physicians’ burnout (measured by the OLBI-subscales exhaustion) considering individual factors and work-home-interaction. The initial response and loss to follow-up was moderate for both genders. In hierarchical multiple regression models the effects were examined separately for males and females. Control variables (age, marital status, number of children younger than six years, working hours), individual variables (job performance-based self-esteem, goal orientation, value congruency), and work-home-interaction (work-home/home-work conflict, work-home/home-work facilitation) were included in the analysis, controlled for baseline exhaustion. Workload was significantly positively associated with exhaustion at follow up in both sexes, and autonomy had a buffering effect on exhaustion only in male physicians.

Lorente Prieto et al. [48] investigated the relationship of job demands and burnout in 274 teachers (57% females) drawn from secondary schools in Spain. Initial response and loss to follow-up were acceptable. The period between the two surveys was only eight months. Exposure was operationalized by job demands (i.e., quantitative overload, mental demands, emotional demands, role ambiguity, and role conflicts). Burnout was measured by the subscale exhaustion of the Spanish version of the MBI-GS. After the stepwise introduction of the different independent variables into the hierarchical regression model and control for baseline exhaustion in the final model (adjusted for age and gender, job demands, job resources, personal resources), only quantitative overload at baseline was significantly related to exhaustion at follow-up. Women felt more exhausted than men, even when controlling for the baseline levels of burnout symptoms. However, the authors did not conduct gender-specific analyses of the relationship between psychosocial working conditions and exhaustion.

The two-wave panel study of Taris et al. [50] examined the effects of job characteristics (job demands, job control) on emotional exhaustion (MBI-GS) in Dutch police officers (n = 5,277; 17% females) during one year. After exclusion of subjects who refused participating at the follow-up investigation (48%) and of subjects with serious burnout symptoms at baseline, 1,000 subjects were randomly selected to be included in the follow-up, of whom 828 participated. As the sampling procedure is well-described and as subjects with serious burnout symptoms were excluded from baseline, the overall study quality was assessed as just acceptable despite of the relatively high loss to follow-up. Using structural equation modeling (SEM) high demands at baseline were related to higher emotional exhaustion at follow-up. In the summary model no significant association was found between job control at baseline and emotional exhaustion. The authors do not report gender-specific analyses.

In a one-year follow-up study, Van Vegchel et al. [52] included 2,255 Swedish human services employees (59% females) of Social Insurance Organizations. Initial response was good (76%) and loss to follow-up was moderate (29%). The primary aim of the study was to examine the moderating effect of both social support and job control on the relationship between quantitative or emotional job demands and burnout, assessed by the subscale emotional exhaustion (MBI). In the hierarchical regression analysis, quantitative demands and emotional demands were separately examined with respect to burnout and its dimensions. The authors found positive associations between quantitative as well as emotional demands with burnout at follow-up. Burnout was negatively associated with control and social support at baseline. Emotional demands had a slightly stronger effect on burnout than quantitative demands. Job control was found to constitute an effect modifier of the relationship between emotional demands and emotional exhaustion. The authors do not report gender-specific analyses of the association between psychosocial working conditions and burnout or emotional exhaustion.

The studies included in our systematic review varied greatly with respect to their population, study design, the number of occupational groups included, the instruments applied to measure exposure and outcomes and in the method of data analysis. Because a sufficient number of studies with comparable exposure measurements (e.g. job content questionnaire JCQ and single items), outcome measures and comparable analytic approaches was lacking, we qualitatively summarized the evidence for an association between the single psychosocial work exposures and burnout.

All six included studies examined the relationship between *work task characteristics* and burnout or its core component emotional exhaustion. In five of these studies [46-48,50,52], the association between high workload / quantitative demands / overload and the development of the burnout core-component emotional exhaustion was examined. All of these studies found a statistically significant increase in emotional exhaustion among persons with high workload or high quantitative demands/ overload. Moreover, statistically significant associations between high emotional demands and emotional exhaustion could be found by van Vegchel et al. [52]. Concerning the influence of low job control on burnout dimensions, we found inconsistent results: While Van Vegchel et al. [52] as well as Janssen and Nijhuis [46] found a significant relationship between low job control or the analogous decreased decision latitude and emotional exhaustion, Langballe et al. [47] revealed a statistically significant relationship between high autonomy and increased emotional exhaustion among male physicians.

Three studies examined the influence of *social conditions* on single burnout dimensions. In two studies low social support predicted increased emotional exhaustion [46,52]. Role conflicts were not found to be associated with burnout subscales [48].

*Work organizational factors* were assessed only in one study [47]. In this study no significant influence of the number of working hours on emotional exhaustion was found for both genders.

Altogether, most studies applying an adequate methodology found a relationship between “poor” psychosocial working conditions and burnout/emotional exhaustion. According to the Oxford Centre for Evidence Based Medicine (CEBM) Levels of Evidence Working Group [56], the level of evidence formally is classified as level 3 because exclusively non-randomized controlled cohort/follow-up studies could be identified.

## Discussion

In our comprehensive systematic review, only six longitudinal studies of adequate methodological quality could be identified that investigated the relationship between psychosocial working conditions (categories: work organization, work task and social conditions) and the development of burnout/emotional exhaustion. We found a relatively consistent association between “unfavorable” psychosocial working conditions (high workload, high quantitative, mental or emotional demands, low social support) and burnout and emotional exhaustion as the core burnout component. The influence of low job control on burnout dimensions was inconsistent. Altogether, with the exception of one study [47] which found that autonomy was a risk factor for later emotional exhaustion among male physicians, we found no statistically significant associations between “favorable” psychosocial working conditions and burnout or emotional exhaustion.

### Strengths and limitations

The main strengths of our research methods were the systematic literature search using a comprehensive search string in two applicable databases (Medline and PsycInfo), the independent appraisal of the retrieved titles and abstracts by two researchers and the independent dual assessment of study quality with consensus-finding to determine the final quality score in the case of discrepancies. Compared to other literature reviews, relatively specific inclusion criteria were defined; therefore cross-sectional studies were excluded from the systematic review to best avoid reverse causality. In addition, studies with inadequate methodological quality were not included in the further appraisal of the evidence (for example cohort studies with a very high loss-to-follow-up or cohort studies that failed to determine if the outcome of interest was present at baseline). Furthermore, only articles published in scientific journals were included. The resulting exclusion of a high number of studies means a loss of information; this is a limitation of our systematic review that should be considered. However, including low quality studies would have introduced considerable bias, particularly “cause-and-effect bias”. Furthermore, the exclusion of low quality-studies might to a certain extent have reduced the potential for publication bias, as the publication chance (particularly) of low-quality studies might have been higher in case of positive findings.

There is no overlap of studies included in our review and the studies included in the above mentioned previous reviews. This is not surprising, as our inclusion criteria differed particularly with respect to the included study populations and study designs (exclusion of cross-sectional studies) and as methodologically poor studies were excluded from our review.

A limitation of our systematic review lies in the difficult differential diagnosis between burnout and clinical psychiatric diagnoses, particularly depressive disorders. We do not know from most of the included studies which proportion of the study probands with burnout actually suffers from a depressive disorder. In fact there is increasing consensus in the scientific community that burnout and depression have to be considered as two separate phenomena, albeit sharing a range of common characteristics [10,11,28,57]. On the other hand, there is obviously a broad area of overlap between burnout and depression [58]. The likelihood of depressive symptoms increases with the severity of burnout (especially in its late and most severe stages), and burnout may progress to clinically manifest depression, but not vice-versa [10,11,59]. Although some studies have examined the pathogenesis of burnout and its link to depression, the relationship between burnout and depression is not yet completely understood [57,59]. Moreover, in clinical practice depressive disorders might sometimes be (mis-) classified as burnout, as burnout is widely considered as a “fashionable diagnosis” [58] that might avoid stigmatization of the (in fact depressive) patients. Only one of the studies included in this review assessed both burnout and depression [45]. When the level of burnout at baseline was adjusted for in the final model, the effect of job strain on depression disappeared in the mentioned study. According to the authors, this result shows “that burnout fully mediated the impact of job strain on depressive symptoms” [45]. The results of our parallel conducted systematic review on the relationship between psychosocial working conditions and depression will be published separately.

The clear dependency of the risk estimates on the composition of the baseline study group points to a fundamental methodological problem: Adjustment for the considered outcome does not necessarily lead to the same results as exclusion of the considered outcome from baseline. However, very few studies would have been left if we had only included studies with exclusion of the considered outcome from baseline. Although this procedure would have been methodologically correct, we therefore decided to also include studies that adjusted for burnout/emotional exhaustion at baseline.

Another limitation of our systematic review lies in the definition of burnout in the included studies: 5 of 6 included studies applied Maslach’s burnout inventory in their operationalization of burnout. It is methodologically problematic that the MBI construct of emotional exhaustion – in contrast to the CBI – predefines exhaustion as due to work: Several MBI emotional exhaustion scale items are closely connected with the psychosocial working conditions, e.g. “I feel emotionally drained from my work”, “I feel tired when I get up in the morning and have to face another day on the job”, and “Working all day is really a strain for me”. Therefore the outcome measure partly includes the “exposure” under study in this review, i.e. “unfavorable working conditions”. Lindeberg et al. [24] rightly point out that in epidemiological studies of associations between working conditions and burnout, an overlap between exposure and outcome inherent in the outcome measure should create potential overestimation of associations. The high OR (22.3) which was found in one of our reviewed studies [45] could at least partly be due to this bias. However, the consequences of this bias might in fact be more complex: When adjusting for baseline burnout/emotional exhaustion or excluding subjects with burnout/emotional exhaustion at baseline, the resulting partial “control” of working conditions might in tendency also lead to an underestimation of burnout risks. Future studies on the relationship between working conditions and emotional exhaustion/burnout should include burnout measures that do not implicitly or explicitly include the working conditions. In case the MBI was applied in a study, at least a subanalysis excluding the “problematic” items should be considered.

A further limitation of our review lies in the insufficient consideration of potential effect modification by gender in the included studies: Most studies solely included gender as a potential confounder, but did not consider gender as a potential effect modificator (by giving separate results for men and women or by introducing interaction terms). Only two included studies analyzed the relationship between psychosocial working conditions and burnout/emotional exhaustion separately for men and women: Langballe et al. [47] found an increased exhaustion risk for high workload among male as well as female physicians; in contrast, the lowered exhaustion risk for high autonomy was restricted to male physicians. Ahola and Hakanen [45] revealed a considerably higher burnout risk for high job strain among men than among women (OR = 22.3 versus 4.0). These authors could in fact find a significant interaction between sex and strain in the model of strain on burnout. The to-date sparse results on gender-specific risks do not yet allow any definite conclusions. Future studies on the relationship between psychosocial working conditions and burnout respectively emotional exhaustion should therefore generally conduct separate analyses for males and females.

### Could specific working conditions be identified as risk factors for burnout/emotional exhaustion?

Based on these six cohort studies, the results of our analysis indicate that particularly “high demands” or increased “job strain” (measured with the JCQ) are a risk factor for emotional exhaustion/burnout. From these results it cannot be concluded that increased “job strain” is the main risk factor for diminished emotional exhaustion/burnout. The fact that positive— and partly statistically significant— associations between increased “job strain” or “high demands” and emotional exhaustion or burnout were repeatedly reported corresponds with the fact that this hypothesis was most frequently examined. On the other hand, the relatively few associations reported among rarely examined factors (i.e. lack of social support at work) do not in any way indicate that these factors are not risk factors for psychological problems. In several large cross-sectional studies applying the comprehensive COPSOQ instrument [60,61], high work-privacy conflicts, high insecurity at work and mobbing were most closely related to burnout; further longitudinal studies should consider these factors.

We would like to point out that strong intercorrelation between workplace factors, as a matter of principle, make the identification of a single psychosocial workplace factor (being associated with an especially high or low risk of burnout) difficult. We therefore regard it as improbable that future research will find “the one” psychosocial work factor that is responsible for emotional exhaustion/burnout. It appears more likely that like other diseases with multifactorial causes, such as musculoskeletal diseases [5] a comprehensive concept for designing working conditions could be especially beneficial for the prevention of occupational psychological diseases.

### Is burnout/emotional exhaustion impaired by objectifiable psychosocial working conditions or (only) by perceived “subjective” strain?

Emotional exhaustion/burnout presumably influence work and how working conditions are perceived. Cross-sectional studies are especially subject to this sort of reverse causation - as well as a muddling of cause and effect. For this reason, cross-sectional studies were excluded from our systematic review. However, this does not imply that the studies that were included observed only “objectifiable” working conditions as risk factors for later emotional exhaustion/burnout. It is imaginable and perhaps even likely, that perceived psychosocial working conditions can (also) act as a stressor that could lead to health problems. Which working conditions are considered psychologically stressful is subject to inter-individual variation. It should be mentioned, that the instruments used by all of the included studies to estimate the psychosocial working conditions (e.g., the demand-control model) primarily depict the perceived and not necessarily the “objectifiable” psychological stress [62]. However Waldenström et al. [63] could find no systematic difference between the self-reported and externally assessed psychosocial working conditions, adjusted for the individual psychological stress level (measured with the GHQ-12) of the study participants. Moreover, it is impossible to conclude from our results that only perceived psychosocial working conditions are a threat to emotional exhaustion/burnout. In a simultaneously conducted systematic review of psychosocial working conditions and depression we could find many more methodologically adequate studies [55]. In this review, studies that used “objective” instruments - independent of the employees’ dispositions - to measure psychosocial workload also found significant associations with the subsequent occurrence of psychological disorders. Therefore both objectifiable occupational stress as well as the felt “subjective” stress might be damaging to mental health.

However, improving working conditions can only influence the “objective” stressors. Which working arrangements are truly effective, can only be determined with intervention studies of excellent methodical quality. We did not find studies directly investigating the effect of psychosocial working conditions on the psychological health. Hence, research needs to devote additional attention to the conducting of intervention studies. Similarly, the practical planning and realization of working conditions should principally be conducted in cooperation with a scientific evaluation. There are many published examples of ambitious intervention programs that ultimately showed no impact on psychosocial work stressors (see, for example, Haukka et al. [64]; Driessen et al. [65]). Identifying approaches that effectively reduce psychosocial work stressors and the consideration of their resulting health effects represent essential future objectives.

Although this analysis of psychosocial working conditions for burnout and emotional exhaustion found numerous thematically relevant studies, very few relevant studies were identified that applied rather adequate scientific methods. Severe selection effects, a possible consequence of high loss-to-follow-up, were one of the most common limitations present in the original studies. We would like to emphasize that even some overall still “methodologically adequate” studies showed a high loss-to-follow-up (particularly [48,50]); therefore, selection bias might have influenced the results. In recent years, various disciplines, such as epidemiology, psychology, and statistics have been developing methods that can help to avoid bias and determine causal associations. Interdisciplinary collaborations and exchanges of ideas and methods could prove advantageous for determining the health consequences of psychosocial work stress. For example, the experiences gained in recent years from research on the effects of psychosocial work stressors on the cardiovascular system [2] could also be applied to research on the psychological effects of psychosocial work stressors. This implies that working conditions that are designed to effectively reduce detrimental work stress may not only prevent psychological diseases but should benefit cardiovascular health as well.

## Conclusion

This systematic review of psychosocial working conditions and burnout and its core component emotional exhaustion found very few methodologically adequate studies. We therefore have to state a strong discrepancy between the high presence of burnout risks in the present public discussion and the low number of corresponding methodologically sound studies. Selection effects, a possible consequence of high loss-to-follow-up, were one of the most common limitations present in the original studies.

Nevertheless, the resulting evidence of our systematic review together with the evidence of a simultaneously conducted review on mental disorders [55] indicates that mental health is related to psychosocial working conditions. Therefore the creation of healthy working conditions appears to be essential for preventing psychological disease. Both objectifiable as well as “subjective” stress might impair mental health. Of course, redesigning working conditions can naturally only influence the “objective” stressors. Identifying effective preventive approaches requires the realization of methodologically impeccable intervention studies. This in mind, additional intervention studies are greatly needed. No one isolated workplace factor could be identified that could serve as the focus of such intervention studies. More probably - analogous to other diseases caused by a multitude of factors - comprehensive multidimensional approaches to designing working conditions may prove to be most effective for preventing psychological diseases. Psychosocial working conditions can not only influence emotional exhaustion/burnout, but also a wide variety of other health outcomes as cardiovascular diseases and musculoskeletal disorders. Therefore the improvement of psychosocial working conditions might not only serve to prevent burnout, but is also a relevant approach to the promotion of public health.

## Competing of interest

The authors declare that they have no competing interests.

## Authors’ contributions

AS, SR and KN developed the methodological concept. SD and FT screened titles and abstracts of the studies and examined the full texts for inclusion. MT and FT performed the data abstraction from the included articles and evaluated the study quality. AS moderated a consensus conference in case of diverging opinions and drafted the manuscript. JH, SR, MT, SD and KN participated in the interpretation of the study results and the drafting of the manuscript. All authors participated in the conduction of the study, read and approved the final manuscript.

## Supplementary Material

Additional file 1 **Gives the Medline via PubMed search strings on the basis of the PEO (D)-criteria (sensitive and specific search string according tob Mattioli et al. **[43]). Click here for file

Additional file 2 Adapted PsycInfo via EBSCO search string on the basis of the PEO (D)-criteria. Click here for file

Additional file 3 **Gives a list of the studies that were excluded based on inadequate quality assessment; the concrete reasons for inadequate quality scores are given by Thinschmidt et al. **[55]. Click here for file
