# Supplementary material for: The role of psychosocial working conditions on burnout and its core component emotional exhaustion – a systematic review
Source: J Occup Med Toxicol. 2014 Mar 14;9:10. doi: 10.1186/1745-6673-9-10 (PMC4233644; doi:10.1186/1745-6673-9-10)
Supplement: Additional file 1 — Gives the Medline via PubMed search strings on the basis of the PEO (D)-criteria (sensitive and specific search string according tob Mattioli et al. [43]). [file 1745-6673-9-10-S1.docx]

Search string Medline

Population 1* (*sensitive search strategy according to Mattioli et al*. [43]):

(occupational diseases [MH] OR occupational exposure [MH] OR occupational exposure* [TW] OR “occupational health” OR “occupational medicine” OR work-related OR working environment [TW] OR at work [TW] OR work environment [TW] OR occupations [MH] OR work [MH] OR workplace* [TW] OR workload OR occupation* OR worke* OR work place* [TW] OR work site* [TW] OR job* [TW] OR occupational groups [MH] OR employment OR worksite* OR industry)

Exposure 1*:

“job strain” OR “mental strain” OR “occupational strain” OR “work strain” or “mental load” OR workload OR work load OR “organisational justice” OR “work stress” OR “job stress” OR psychosocial work* OR “organizational justice” OR “organisational injustice” OR “organizational injustice” OR “time pressure” OR “pressure of time” OR harassment OR (effort AND reward) OR demand* OR “shift work” OR mobbing OR bullying OR leadership OR “social relations” OR “social support” OR “job insecurity” OR downsiz*

Population 2* (*specific search strategy according to Mattioli et al*. (*2010*)):

(occupational diseases [MH] OR occupational exposure [MH] OR occupational medicine [MH] OR occupational risk [TW] OR occupational hazard [TW] OR (industry [MeSH Terms] mortality [SH]) OR occupational group* [TW] OR work-related OR occupational air pollutants [MH] OR working environment [TW])

Exposure 2*:

(stress OR conflict OR support OR climate)

Outcome*:

mental disorders [MeSH] OR burnout OR “mental health” OR anxiety OR depress* OR emotional disorder* OR exhaust* OR psychosomatic OR somatoform*

Study Design*:

“randomized controlled trial” OR RCT OR intervention OR Epidemiologic studies [MeSH] OR Case control [tw] OR cohort study [tw] OR cohort studies [tw] OR Cohort analy*[tw] OR follow up study [tw] or follow up studies [tw] OR observational study [tw] or observational studies [tw] OR “prospective study” OR Longitudinal [tw] OR Retrospective [tw] NOT therapy NOT ((animals [Mesh:noexp]) NOT (humans [Mesh]))

*Search terms were combined as follows: ((Population 1 AND Exposure 1) OR (Population 2 AND Exposure 2)) AND Outcome AND Study Design
