# Supplementary material for: The role of psychosocial working conditions on burnout and its core component emotional exhaustion – a systematic review
Source: J Occup Med Toxicol. 2014 Mar 14;9:10. doi: 10.1186/1745-6673-9-10 (PMC4233644; doi:10.1186/1745-6673-9-10)
Supplement: Additional file 2 — Adapted PsycInfo via EBSCO search string on the basis of the PEO (D)-criteria. [file 1745-6673-9-10-S2.docx]

Search string PsycInfo*.

Population:

occupation* OR employ* OR work* OR job

Exposure:

(TX “job strain”) OR (TX “mental strain”) OR (TX “occupational strain”) OR (TX “work strain”) or (TX “mental load”) OR (TX workload) OR (TX “work load”) OR (TX (work N3 stress*)) OR (TX “job stress”) OR (TX “organi?ational justice”) OR (TX “organi?ational injustice”) OR (TX “job insecurity”) OR (TX (time N4 pressure)) OR (TX conflict*) OR (TX support) OR (TX harassment) OR (TX climate) OR (TX downsiz*) OR (TX (effort AND reward)) OR (TX demand*) OR (TX (shift N3 work*)) OR (TX mobbing) OR (TX bullying) OR (TX leadership) OR (TX “social relations”) OR (TX “social support”)

Outcome:

(SU “mental disorders”) OR (SU “adjustment disorders”) OR (SU “affective disorders”) OR (SU “anxiety disorders”) OR (SU “work related illnesses”) OR (SU “occupational stress”) OR (emotional N2 disorder*) OR (SU “somatoform disorder”) OR burnout

Study Design:

((TX “randomized controlled trial”) OR (TX RCT) OR (TX intervention) OR (TX “case control”) OR (TX (cohort W2 stud*)) OR (TX (cohort W2 analy*)) OR (TX (follow W3 stud*)) OR (TX followup) OR (TX (observational W2 stud*)) OR (TX “prospective study”) OR (TX longitudinal) OR (TX “retrospective”)) NOT (“cross-sectional” OR “prevalence study” OR therapy).

* search term were combined as follows: Population AND Exposure AND Outcome AND Study design.
