# Supplementary material for: The role of psychosocial working conditions on burnout and its core component emotional exhaustion – a systematic review
Source: J Occup Med Toxicol. 2014 Mar 14;9:10. doi: 10.1186/1745-6673-9-10 (PMC4233644; doi:10.1186/1745-6673-9-10)
Supplement: Additional file 3 — Gives a list of the studies that were excluded based on inadequate quality assessment; the concrete reasons for inadequate quality scores are given by Thinschmidt et al. [55]. [file 1745-6673-9-10-S3.docx]

Angelo RP, Chambel MJ. The reciprocal relationship between work characteristics and employee burnout and engagement: A longitudinal study of firefighters. *Stress Health* 2013, 2013 Oct 10. doi: 10.1002/smi.2532.

Bakker AB, Schaufeli WB, Sixma HJ, Bosveld W, van Dierendonck D. Patient demands, lack of reciprocity, and burnout: A five-year longitudinal study among general practitioners. *J Organiz Behav* 2000, 21:425–441.

Barton J, Folkard S, Smith L, Poole CJ. Effects on health of a change from a delaying to an advancing shift system. *Occup Environ Med* 1994, 51:749–755.

Blau G, Ward-Cook K. A brief note on further investigating correlates of work exhaustion for medical technologists. *J Allied Health* 2006, 35:e6-e21.

Blom V. Contingent self-esteem, stressors and burnout in working women and men. *Work* 2012, 43(2):123–131.

Borritz M, Bultmann U, Rugulies R, Christensen KB, Villadsen E, Kristensen TS. Psychosocial work characteristics as predictors for burnout: findings from 3-year follow up of the PUMA Study. *J Occup Environ Med* / *American College Occup Environ Med* 2005, 47:1015–1025.

Bourbonnais R, Brisson C, Vezina M. Long-term effects of an intervention on psychosocial work factors among healthcare professionals in a hospital setting. *Occup Environ Med* 2010, 68(7):479–86.

Brauchli R, Schaufeli WB, Jenny GJ, Füllemann D, Bauer GF. Disentangling stability and change in job resources, job demands, and employee well-being—A three-wave study on the job-demands resources model. *J Vocat Behav* 2013, 83(2):117–129.

Burke RJ. Work experiences and psychological well-being of former hospital-based nurses now employed elsewhere. *Psychol Rep* 2002, 91:1059–64.

Burke RJ, Greenglass ER. A longitudinal examination of the Cherniss model of psychological burnout. *Soc Sci Med* 1995, 40:1357–63.

Burke RJ, Greenglass ER, Schwarzer R. Predicting teacher burnout over time: Effects of work stress, social support, and self-doubts on burnout and its consequences. *Anxiety stress coping* 1996, 9:261–275.

Büssing A, Glaser J. Work stressors in nursing in the course of redesign: Implications for burnout and interactional stress. *Eur J Work Organiz Psychol* 1999, 8:401–426.

Demerouti E, Bakker AB, Bulters AJ. The loss spiral of work pressure, work-home interference and exhaustion: Reciprocal relations in a three-wave study. *J Vocat Behav* 2004, 64:131–149.

Demerouti E, Le Blanc PM, Bakker AB, Schaufeli WB, Hox J. Present but sick: A three-wave study on job demands, presenteeism and burnout. *Career Develop Internat* 2009, 14:50–68.

Devereux JM, Hastings RP, Noone SJ, Firth A, Totsika V. Social support and coping as mediators or moderators of the impact of work stressors on burnout in intellectual disability support staff. *Res Dev Disabil* 2009, 30:367–377.

Dignam JT, West SG. Social support in the workplace: tests of six theoretical models. *Am J Community Psychol* 1988, 16:701–724.

Fernet C, Gagné M, Austin S. When does quality of relationships with coworkers predict burnout over time? The moderating role of work motivation. *J Organiz Behav* 2010, 31:1163–1180.

Feuerhahn N, Bellingrath S, Kudielka BM. The interplay of matching and non-matching job demands and resources on emotional exhaustion among teachers. *Appl Psychol Health Well Being* 2013, 5(2):171–192.

Fong CM. A longitudinal study of the relationships between overload, social support, and burnout among nursing educators. *J Nurs Educ* 1993, 32:24–29.

Gelsema TI, Van der Doef M, Maes S, Janssen M, Akerboom S, Verhoeven C. A longitudinal study of job stress in the nursing profession: causes and consequences. *J Nurs Manag* 2006, 14:289–299.

Greenglass ER, Burke RJ, Konarski R. Components of burnout, resources, and gender-related differences. *J Appl Soc Psychol* 1998, 28:1088–1106.

Hakanen JJ, Schaufeli WB, Ahola K. The Job Demands-Resources model: A three-year cross-lagged study of burnout, depression, commitment, and work engagement. *Work Stress* 2008, 22:224–241.

Hall GB, Dollard MF, Tuckey MR, Winefield AH, Thompson BM. Job demands, work–family conflict, and emotional exhaustion in police officers: A longitudinal test of competing theories. *J Occup Organiz Psychol* 2010, 83:237–250.

Hansson A, Vingård E, Arnetz BB, Anderzén I. Organizational change, health, and sick leave among health care employees: A longitudinal study measuring stress markers, individual, and work site factors. *Work Stress* 2008, 22:69–80.

Hillhouse JJ, Adler CM, Walters DN. A simple model of stress, burnout and symptomatology in medical residents: A longitudinal study. *Psychol Health Med* 2000, 5:63–73.

Hornung S, Weigl M, Glaser J, Angerer P. Is it so bad or am I so tired? cross-lagged relationships between job stressors and emotional exhaustion of hospital physicians. *J Person Psychol* 2013, 12(3):124–131.

Houkes I, Janssen PP, De Jonge J, Bakker AB. Personality, work characteristics, and employee well-being: a longitudinal analysis of additive and moderating effects. *J Occup Health Psychol* 2003, 8:20–38.

Houkes I, Janssen PPM, de Jonge J, Bakker AB. Specific determinants of intrinsic work motivation, emotional exhaustion and turnover intention: A multisample longitudinal study. *J Occup Organiz Psychol* 2003, 76:427–450.

Kirk SA, Koeske GF, Koeske RD. Changes in health and job attitudes of case managers providing intensive services. *Hosp Community Psychiatry* 1993, 44:168–173.

Leiter MP, Durup MJ. Work, home, and in-between: A longitudinal study of spillover. *J Appl Behav Sci* 1996, 32:29–47.

Liljegren M, Ekberg K. The associations between perceived distributive, procedural, and interactional organizational justice, self-rated health and burnout. *Work* 2009, 33:43–51.

Liljegren M, Ekberg K. The longitudinal relationship between job mobility, perceived organizational justice, and health. *BMC Public Health* 2008, 19:164.

Lizano EL, Mor Barak ME. Workplace demands and resources as antecedents of job burnout among public child welfare workers: A longitudinal study. *Children and Youth Services Review* 2012, 34(9):1769–1776.

Lökk CT, Arnetz BB. Impact of management change and an intervention program on health care personnel. *Psychother Psychosom* 2000, 69:79–85.

Magnusson Hanson LL, Theorell T, Oxenstierna G, Hyde M, Westerlund H. Demand, control and social climate as predictors of emotional exhaustion symptoms in working Swedish men and women. *Scand J Public Health* 2008, 36:737–743.

Mäkikangas A, Kinnunen U. Psychosocial work stressors and well-being: Self-esteem and optimism as moderators in a one-year longitudinal sample. *Pers Individ Dif* 2003, 35:537–557.

Mattila P, Elo A, Kuosma, E, Kylä-Setälä E. Effect of a participative work conference on psychosocial work environment and well-being. *Eur J Work Organiz Psychol* 2006, 15:459–476.

Mauno S, Kinnunen U. Job insecurity and well-being: A longitudinal study among male and female employees in Finland. *Community Work Family* 1999, 2:147–171.

Melamed S, Armon G, Shirom A, Shapira I. Exploring the reciprocal causal relationship between job strain and burnout: A longitudinal study of apparently healthy employed persons. *Stress Health* 2011, 27(4):272–281.

Mirvis DM, Graney MJ, Kilpatrick AO. Burnout among leaders of Department of Veterans Affairs medical centers: contributing factors as determined by a longitudinal study. *J Health Hum Serv Adm* 1999, 21:390–412.

Peiró JM, Gonzalez-Romá V, Tordera N, Mañas MA. Does role stress predict burnout over time among health care professionals? *Psychol Health* 2001, 16:511–525.

Quested E, Duda JL. Antecedents of burnout among elite dancers: A longitudinal test of basic needs theory. *Psychol Sport Exerc* 2011, 12(2):159–167.

Ramarajan L, Barsade SG, Burack OR. The influence of organizational respect on emotional exhaustion in the human services. *J Posit Psychol* 2008, 3:4–18.

Schaufeli WB, Bakker AB, Van Rhenen W. How changes in job demands and resources predict burnout, work engagement and sickness absenteeism. *J Organiz Behav* 2009, 30:893–917.

Schaufeli WB, Maassen GH, Bakker AB, Sixma HJ. Stability and change in burnout: A 10-year follow-up study among primary care physicians. *J Occup Organ Psychol* 2011, 84(2):248–267.

Schmitz GS. Kann Selbstwirksamkeiterwartung Lehrer vor Burnout schützen? Eine Längsschnittstudie in zehn Bundesländern. *Psychologie in Erziehung und Unterricht* 2000, 48:49–67.

Schwarzer R, Hallum S. Perceived teacher self-efficacy as a predictor of job stress and burnout. *Appl Psychol*: *Internat Rev* 2008, 57:152–171.

Sonnentag S, Binnewies C, Mojza EJ. Staying well and engaged when demands are high: The role of psychological detachment. *J Appl Psychol* 2010, 95:965–976.

Sørgaard KW, Ryan P, Hill R, Dawson I. Sources of stress and burnout in acute psychiatric care: Inpatient vs. community staff. *Soc Psychiatry Psychiatr Epidemiol* 2007, 42:794–802.

Stahl JE, Egan MT, Goldman JM, Tenney D, Wiklund RA, Sandberg WS, Gazelle S, Rattner DW. Introducing new technology into the operating room: measuring the impact on job performance and satisfaction. *Surgery* 2005, 137:518–526.

Sterud T, Hem E, Lau B, Ekeberg O. A comparison of general and ambulance specific stressors: Predictors of job satisfaction and health problems in a nationwide one-year follow-up study of norwegian ambulance personnel. *J Occup Med Toxicol* 2011, 6(1):10-6673-6-10.

Taris TW, Peeters MC, Le Blanc PM, Schreurs PJ, Schaufeli WB. From inequity to burnout: the role of job stress. *J Occup Health Psychol* 2001, 6:303–323.

Taylor C, Graham J, Potts HWW, Richards MA, Ramirez AJ. Changes in mental health of UK hospital consultants since the mid-1990s. *Lancet* 2005, 366:742–744.

Tzischinsky O, Zohar D, Epstein R, Chillag N, Lavie P. Daily and yearly burnout symptoms in Israeli shift work residents. *J Hum Ergol* 2001, 30:357–362.

Van de Ven B, van den Tooren M, Vlerick P. Emotional job resources and emotional support seeking as moderators of the relation between emotional job demands and emotional exhaustion: A two-wave panel study. *J Occup Health Psychol* 2013, 18(1):1–8.

Van der Ploeg E, Kleber RJ. Acute and chronic job stressors among ambulance personnel: predictors of health symptoms. *Occup Environ Med* 2003, 60:i40-6.

Wade DC, Cooley E, Savicki V. A longitudinal study of burnout. *Child Youth Serv Rev* 1986, 8:161–173 Westman M, Bakker AB, Roziner I, Sonnentag S. Crossover of job demands and emotional exhaustion within teams: a longitudinal multilevel study. *Anxiety Stress Coping* 2011, 18:1–17.

Wolpin J, Burke RJ, Greenglass ER. Is job satisfaction an antecedent or a consequence of psychology burnout? *Hum Relat* 1991, 44:193–209.
